# Understanding of Preeclampsia Risk Factors in Large Language Models Compared with a Validated Competing-Risks Model

**DOI:** 10.3390/diagnostics16152340

**Published:** 2026-07-26

**Authors:** Alexandra-Elena Cristofor, Oriana-Maria Onicescu, Denisa-Oana Zelinschi, Alexandra Ursache, Alexandru Carauleanu, Dragos Nemescu

**Affiliations:** 1 Mother and Child Department, Grigore T. Popa University of Medicine and Pharmacy Iasi, 16 Universitatii Str, 700115 Iasi, Romania; dr.acristofor@gmail.com (A.-E.C.); denisa-oana.zelinschi@umfiasi.ro (D.-O.Z.); ale.carauleanu@umfiasi.ro (A.C.); dragos.nemescu@umfiasi.ro (D.N.); 2 Faculty of Computer Science, Alexandru Ioan Cuza University of Iasi, 700506 Iasi, Romania; oriania.oniciuc@info.uaic.ro

**Keywords:** preeclampsia prediction, competing-risks model, large language models, explainable artificial intelligence, Local Interpretable Model-Agnostic Explanations

## Abstract

**Background:** First-trimester screening for preterm preeclampsia relies on validated competing-risks models integrating maternal characteristics with biochemical and biophysical markers to generate individualized risk estimates. Large language models (LLMs) are increasingly used for pregnancy-related health information, yet their alignment with established clinical risk models remains unclear. **Objective:** to perform an exploratory, local perturbation-based assessment of how LLM-generated numerical risk estimates reproduce the direction and relative magnitude of established preeclampsia predictor effects compared with the Fetal Medicine Foundation (FMF) competing-risks model. **Methods:** A total of 129 synthetic clinical scenarios were generated from a low-risk reference pregnancy using a one-factor-at-a-time perturbation approach across 16 risk factors represented as 22 predictors. Eight LLMs (Claude, GPT, DeepSeek, Gemini, Copilot, Meta, Mistral, and Grok) estimated the probability of preeclampsia requiring delivery before 37 weeks. Corresponding risks were calculated using the FMF model. Model behavior was analyzed in logit space using a local perturbation-based modeling approach inspired by Local Interpretable Model-Agnostic Explanations (LIME) to derive feature-effect coefficients. Agreement with the FMF model was assessed using correlation, directional concordance, cosine similarity, and normalized root mean squared error, summarized using an exploratory composite score. **Results:** The FMF model identified mean arterial pressure, placental growth factor, parity, uterine artery pulsatility index, and chronic hypertension as dominant predictors. Alignment between LLM outputs and the FMF model was heterogeneous, with composite scores ranging from 0.59 to 0.82. Models with higher descriptive scores preserved predictor directionality (up to 90.9%) and rank ordering, but agreement in magnitude and scaling was limited (R^2^: 0.44–0.59). Intermediate models showed preserved directionality with reduced magnitude agreement, while lower-scoring models demonstrated more frequent sign inconsistencies and minimal variance explained. **Conclusions:** LLMs demonstrated partial, prompt-specific alignment with the FMF model in this local perturbation analysis, particularly for predictor direction and relative importance, but did not consistently reproduce quantitative effect sizes. This approach was intended to characterize local model behavior around a predefined reference case rather than evaluate clinically realistic combinations of interacting risk factors or global clinical prediction performance. Given the evolving nature of LLMs, ongoing reassessment using standardized approaches is required.

## 1. Introduction

Preeclampsia is a significant multisystem disorder of pregnancy, recognized as a major contributor to maternal and perinatal morbidity and mortality on a global scale [1]. Current epidemiological studies report a worldwide pooled prevalence of 4.43%, though this rate shows considerable variability depending on the diagnostic criteria employed [2]. The condition is defined by the new onset of hypertension after 20 weeks of gestation, frequently accompanied by proteinuria or evidence of end-organ dysfunction [3].

In clinical settings, classification of this condition is based on gestational age at delivery: preterm preeclampsia refers to cases where delivery occurs before 37 weeks’ gestation, whereas term preeclampsia applies to deliveries at or beyond 37 weeks [4]. This classification is important because preterm preeclampsia is associated with significantly higher risks of adverse maternal and fetal outcomes compared to term preeclampsia [5].

Traditional screening for preeclampsia has historically relied on identifying clinical risk factors from maternal history [3,6,7]. Modern preeclampsia screening is based on specialized models that combine maternal characteristics with biophysical and biochemical tests [4,8,9]. The Fetal Medicine Foundation (FMF) competing-risks model is the most widely validated approach, utilizing Bayes’ theorem to combine a priori risk from maternal factors with biomarkers measured at 11–13 weeks of gestation, and treating gestational age at delivery as a continuous variable. The biomarkers include mean arterial pressure (MAP), uterine artery pulsatility index (UtA-PI), and placental growth factor (PlGF) [8,9].

In recent years, large language models (LLMs) have become increasingly prevalent in healthcare [10,11]. Trained on vast text data, these models can generate and interpret natural language, enabling applications in medical documentation, patient communication, and clinical information delivery. Their accessibility has prompted many people to use LLMs for health advice, including guidance on complex conditions like preeclampsia [12,13].

However, LLMs can produce confident and highly plausible responses that may not accurately reflect clinical evidence [14,15], and substantial variability exists across models due to differences in architecture and training data [16].

Prior research evaluating LLMs in other medical domains, such as endometriosis, has indicated that they often provide “mostly correct but inadequate” responses, showing better performance in describing symptoms and pathophysiology compared to nuanced treatment or recurrence information [17].

Because LLMs operate as complex “black-box” models, it is difficult for clinicians to understand how they arrive at specific risk predictions. Whether LLMs internally reproduce the structure and relative weighting of established preeclampsia risk models remains unknown.

### Objective

To quantify, in an exploratory local perturbation setting, the extent to which contemporary LLMs, without task-specific training, reproduce the direction and relative magnitude of established preeclampsia predictors, including maternal characteristics and first-trimester biophysical and biochemical markers, compared with the FMF-validated competing-risks model.

## 2. Materials and Methods

This study utilized established predictors embedded in the FMF competing-risks model for preeclampsia screening at 11–13 weeks’ gestation [8,9]. These included maternal demographic characteristics, medical and obstetric history, and first-trimester biophysical and biochemical markers: maternal age, race, height, weight, conception type, family history of preeclampsia, chronic hypertension, diabetes mellitus, systemic lupus erythematosus or antiphospholipid syndrome, parity, previous preeclampsia, interpregnancy interval, previous gestational age at delivery, mean arterial pressure (multiples of the median, MoM), mean UtA-PI (MoM), pregnancy-associated plasma protein-A (PAPPA) (MoM), and PlGF (MoM). The FMF competing-risks model was used as a validated clinical reference standard for structural comparison, because of its extensive external validation and widespread clinical adoption.

### 2.1. Clinical Scenario Generation and Model Querying

This study used only synthetic clinical scenarios and involved no patient data. We defined a low-risk reference pregnancy, characterized by median values for continuous variables and the lowest-risk categories for categorical variables. From this reference case, synthetic clinical scenarios were generated using a one-factor-at-a-time perturbation approach. Each risk factor was systematically varied across a predefined clinically plausible range while all other predictors were held constant at reference values. Continuous predictors were sampled across their distribution (1st–99th percentiles), with additional values included at clinically relevant extremes [8,9].

This approach resulted in a total of 129 scenarios, designed to characterize local predictor effects in the neighborhood of a predefined reference pregnancy. This design deliberately isolates the marginal effect of each predictor and does not represent all possible combinations of predictors. It allows controlled estimation of local predictor effects and should be interpreted as a perturbation-based model behavior study rather than a simulation of clinically realistic patient populations, in which multiple risk factors frequently coexist and interact. Finally, these structured tabular scenarios were converted into standardized English-language clinical narratives to ensure compatibility with LLM input requirements.

Each scenario followed a fixed template describing the clinical, demographic, biophysical, and biochemical characteristics of the pregnancy. The following are the clinical, demographic, biophysical, and biochemical characteristics of a [x]-year-old pregnant woman. She is [white/black/east asian/south asian/mixed], has [x] cm height and a weight of [x] kg. Her conception was [spontaneous/by IVF]. She [reports/reports no] family history of preeclampsia. She [has/does not have] chronic hypertension, [has/does not have] diabetes mellitus, and [has/does not have] systemic lupus erythematosus or antiphospholipid syndrome. She is [nullipara/parous without previous preeclampsia/parous with previous preeclampsia]; the interval between pregnancies is [x] years; and the last pregnancy ended at [x] gestational weeks. At first-trimester screening at 11 to 13 gestational weeks, her mean arterial pressure MoM was [x], her mean uterine arteries PI MoM was [x], her PLGF MoM was [x], and her PAPPA MoM was [x].

Each clinical scenario was submitted to multiple LLMs – Claude (Opus 4.5), Copilot (v2025), DeepSeek (V2, DeepThink), Gemini 3 Pro, GPT-5.2, Grok 4 Expert, Meta (Llama 4), and Mistral (Large 2) – which were prompted to estimate the probability of preeclampsia requiring delivery before 37 weeks’ gestation.

All models were queried in October 2025. Models were accessed through their consumer web interface using default provider settings. Temperature, sampling parameters, reasoning-mode controls, and backend version details were not known, nor uniformly available across providers. Each synthetic scenario was submitted once using an identical prompt template. Therefore, the analysis reflects model behavior under a specific prompt, date, access route, and default configuration, and does not quantify within-model stochastic variability or session-to-session reproducibility. No clinical plausibility filter, calibration adjustment, or post hoc correction was applied to the extracted probabilities beyond conversion to logit space for local surrogate modeling.

Raw prompts, synthetic scenarios, and analysis code are provided in the Supplementary Materials to support reproducibility of the reported analyses.

For the same scenarios, preeclampsia risk was independently calculated using the FMF competing-risks model [8,9].

### 2.2. Explainability Analysis

To analyze model behavior, we employed a local analysis approach inspired by Local Interpretable Model-Agnostic Explanations (LIME) [18]. Unlike conventional LIME implementations that generate perturbations from an empirical feature distribution, our analysis used a predefined set of synthetic one-factor-at-a-time perturbations around a clinically defined reference pregnancy. LIME approximates model behavior locally by fitting an interpretable surrogate model in the neighborhood of a given input, rather than attempting to derive a global representation. In this study, LIME was used primarily as a sparse local surrogate modeling framework rather than as a conventional perturbation-generation method.

In our analysis, a low-risk reference pregnancy served as the index case, and the synthetic clinical scenarios generated through one-factor-at-a-time perturbation defined the local neighborhood. This design enabled controlled estimation of the marginal effect of individual predictors on model output while minimizing confounding from higher-order interactions. Consequently, the derived coefficients should be interpreted as local first-order approximations around the selected reference pregnancy and not as global representations of model behavior. Sparse local surrogate models were fitted using a K-LASSO procedure, with the number of retained predictors fixed a priori to ensure comparability across models and to preserve clinical interpretability. Predictor coefficients were derived in logit space, such that positive coefficients indicate increased predicted risk, and negative coefficients indicate decreased risk. Coefficient magnitude reflects the relative contribution of each predictor within the local approximation.

For local surrogate analysis, the 16 clinical and biochemical risk factors included in the FMF competing-risks model were represented as 22 predictors (Supplementary Table S1). This expansion reflects the encoding of categorical variables as binary indicators (one-hot encoding, with reference categories omitted) and the separation of clinically distinct states into independent predictors, while continuous variables were retained as single features. This representation ensured compatibility with the LIME framework and enabled direct comparison of feature-effect coefficients across models.

Positive coefficients indicated higher predicted preeclampsia risk, while negative coefficients suggested lower risk. Coefficient size ranked predictor importance for each individual prediction. Separate local analyses were performed for each model, allowing direct comparison of feature-effect coefficients between LLMs and the FMF competing-risks model.

### 2.3. FMF Fidelity Assessment

Local analysis-derived feature-effect coefficients were used to compare each LLM with the FMF competing-risks model, which served as the clinical reference standard. For each model, coefficients were evaluated against the corresponding FMF coefficients to assess alignment with established preeclampsia risk factors. All analyses included the same set of predictors, identically encoded across models, ensuring direct comparability.

Agreement between LLM-derived and FMF feature-effect coefficients was assessed using multiple complementary metrics, each capturing a distinct aspect of structural fidelity. Pearson correlation quantifies overall linear association between coefficient vectors, reflecting concordance in the relative ordering and direction of predictor effects, independent of scale; however, it does not capture nonlinear relationships and is sensitive to the small number of predictors. Spearman correlation was therefore reported descriptively as a rank-based complement. Directionality concordance measured the proportion of predictors for which both models assigned coefficients of the same sign (for |coefficient| > 10^−6^), capturing agreement in whether individual predictors increase or decrease risk. Magnitude-related agreement was evaluated using two complementary measures. Cosine similarity quantified alignment in the orientation of the coefficient vectors in feature space, emphasizing consistency in the relative contribution of predictors, irrespective of absolute magnitude. In parallel, normalized root mean squared error (NRMSE) assessed absolute differences in coefficient magnitude; for interpretability, NRMSE was transformed into a bounded similarity metric using 1/(1 + NRMSE), such that higher values indicate closer agreement.

To further characterize agreement in predictor scaling, a feature-effect coefficient of determination (FMF feature-effect R^2^) was computed. For each model, LLM-derived coefficients were regressed against the corresponding FMF coefficients across all predictors, and R^2^ was calculated as the proportion of variance in the FMF coefficients explained by the LLM-derived coefficients. This metric captures agreement in both magnitude and scaling of predictor effects, complementing correlation-based measures that are invariant to linear rescaling [19]. Raw negative R^2^ values were retained and reported because they indicate worse-than-mean explanatory performance; for construction of the bounded composite score only, negative R^2^ values were truncated to zero.

To provide a summary measure across these dimensions, an overall FMF Fidelity Score (range 0–1) was calculated as a weighted composite of Pearson correlation (0.30), directionality concordance (0.20), cosine similarity (0.15), NRMSE-based similarity (0.15), and feature-effect R^2^ (0.20). These weights were specified a priori on conceptual grounds to emphasize rank/order agreement and directionality while still incorporating magnitude and variance-explained components. The composite score is not a validated clinical metric and should be interpreted only as an exploratory descriptive summary. Individual components are therefore reported alongside the composite score to allow transparent interpretation. Higher scores indicate closer alignment with the FMF model’s underlying risk structure.

Agreement in dominant predictors was further assessed using the overlap of the top eight features, defined as the number and proportion of shared predictors among the largest coefficients for each model compared with the FMF reference. This measure was reported descriptively and was not included in the composite score.

All analyses were implemented in Python 3.12, using standard scientific libraries, including *numpy*, *pandas*, and *scikit-learn*, with correlation and variance-based measures computed using functions such as pearsonr, cosine_similarity, mean_squared_error, and r2_score.

## 3. Results

**Figure 1:**
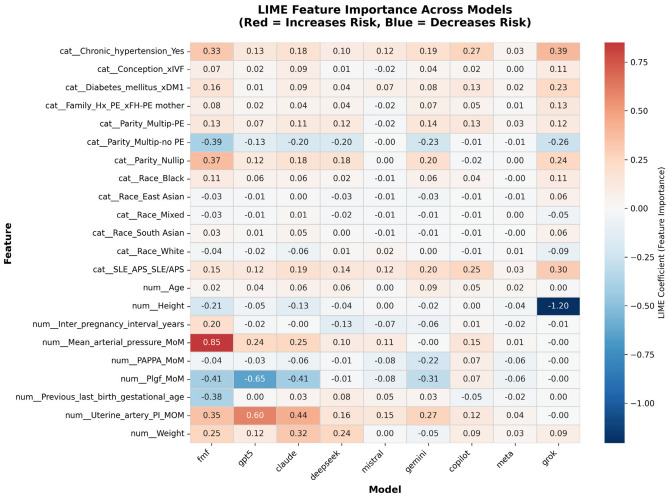
Heatmap of LIME-derived feature-effect coefficients for the FMF model and eight LLMs across 22 preeclampsia predictors. Positive coefficients (red) indicate risk-increasing effects; negative coefficients (blue) indicate protective effects. Color intensity reflects coefficient magnitude.

Local surrogate analysis of the FMF competing-risks model delineated the relative contribution of 22 predictors to the risk of preeclampsia requiring delivery before 37 weeks (Figure 1). Within the FMF reference model, the ten most influential predictors, ranked by decreasing absolute coefficient magnitude, were mean arterial pressure (MAP) MoM (0.85), placental growth factor (PlGF) MoM (−0.41), multiparity without previous preeclampsia (−0.39), previous gestational age at delivery (−0.38), nulliparity (0.37), uterine artery pulsatility index (UtA-PI) MoM (0.35), chronic hypertension (0.33), maternal weight (0.25), maternal height (−0.21), and interpregnancy interval (0.20).

The exploratory composite FMF Fidelity Score demonstrated variability among the eight large language models in their alignment with the FMF competing-risks model (Table 1). Descriptively, the highest scores were observed for Claude (0.815) and GPT-5 (0.778), followed by DeepSeek (0.697), Gemini (0.675), and Copilot (0.666). Meta (0.640), Mistral (0.588), and Grok (0.587) showed lower overall scores. Because the composite score represents an aggregation of multiple agreement metrics, individual components are reported to allow detailed interpretation.

Correlation-based agreement was moderate overall (Table 1). Pearson correlation between local surrogate analysis-derived feature effects and FMF coefficients ranged from 0.78 (Claude) and 0.68 (GPT-5) to 0.39 (Grok). Spearman rank-order correlations were comparatively stronger for the top-performing models, with Claude achieving ρ = 0.86 and GPT-5 achieving ρ = 0.83, indicating relatively consistent ranking of predictor importance despite differences in absolute magnitude.

Directional agreement (risk-increasing vs. risk-decreasing sign) was generally high but was variable across models (Table 1). GPT-5 achieved the highest sign concordance at 90.9% (20/22 predictors), followed by DeepSeek at 86.4% (19/22) and Claude at 81.8% (18/22). In contrast, Mistral showed only 50.0% (11/22) sign concordance, consistent with near-random directional agreement.

Cosine similarity between LLM and FMF coefficient vectors was highest for Claude (0.80) and moderate for GPT-5 (0.69), declining to 0.39 for Grok. Magnitude fidelity assessed via NRMSE was best for Claude (0.14) and progressively worsened across lower-ranked models (up to 0.26 for Grok). The proportion of variance explained in FMF feature-effect coefficients (R^2^) remained limited, including for top-performing models: Claude achieved R^2^ = 0.59 and GPT-5 achieved R^2^ = 0.44, indicating partial but incomplete agreement in predictor magnitude and scaling. Lower-ranked models showed minimal or no explanatory power, with Meta achieving R^2^ = 0.04 and Grok yielding a negative R^2^ (truncated to 0), indicating worse-than-baseline agreement relative to the FMF mean coefficient, consistent with poor alignment in coefficient structure.

Overall, these findings indicate moderate-to-high agreement in selected dimensions of predictor structure, particularly for directionality and rank ordering among higher-performing models, but more limited agreement in the magnitude and scaling of predictor effects.

The degree of overlap among the top eight predictors (Table 1) was greatest between Claude and GPT-5 (7 out of 8), moderate for DeepSeek and Gemini (5 out of 8), and lowest for Grok (3 out of 8).

**Figure 2:**
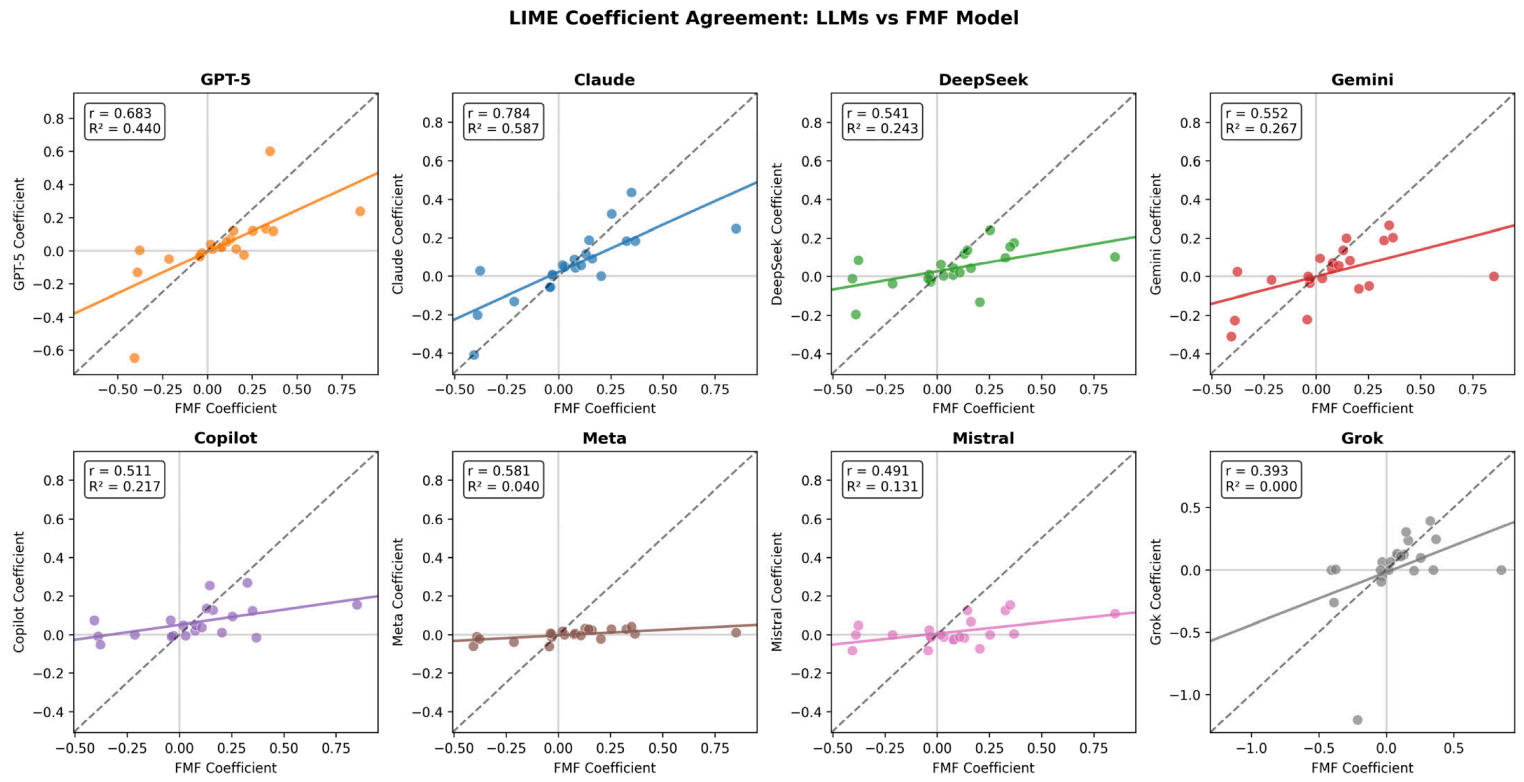
Agreement between LLM and FMF feature-effect coefficients for preeclampsia prediction. Each point represents one of 22 predictors. Dashed line indicates perfect agreement; solid line shows regression fit. Pearson correlation (r) and variance explained (R^2^) are displayed for each model. The R^2^ values shown in each panel correspond to the FMF feature-effect R^2^ reported in Table 1 and quantify variance explained in FMF feature-effect coefficients by the corresponding LLM coefficients.

Figure 2 illustrates the relationship between LLM-derived and FMF feature-effect coefficients, highlighting differences in both direction and magnitude across models.

**Figure 3:**
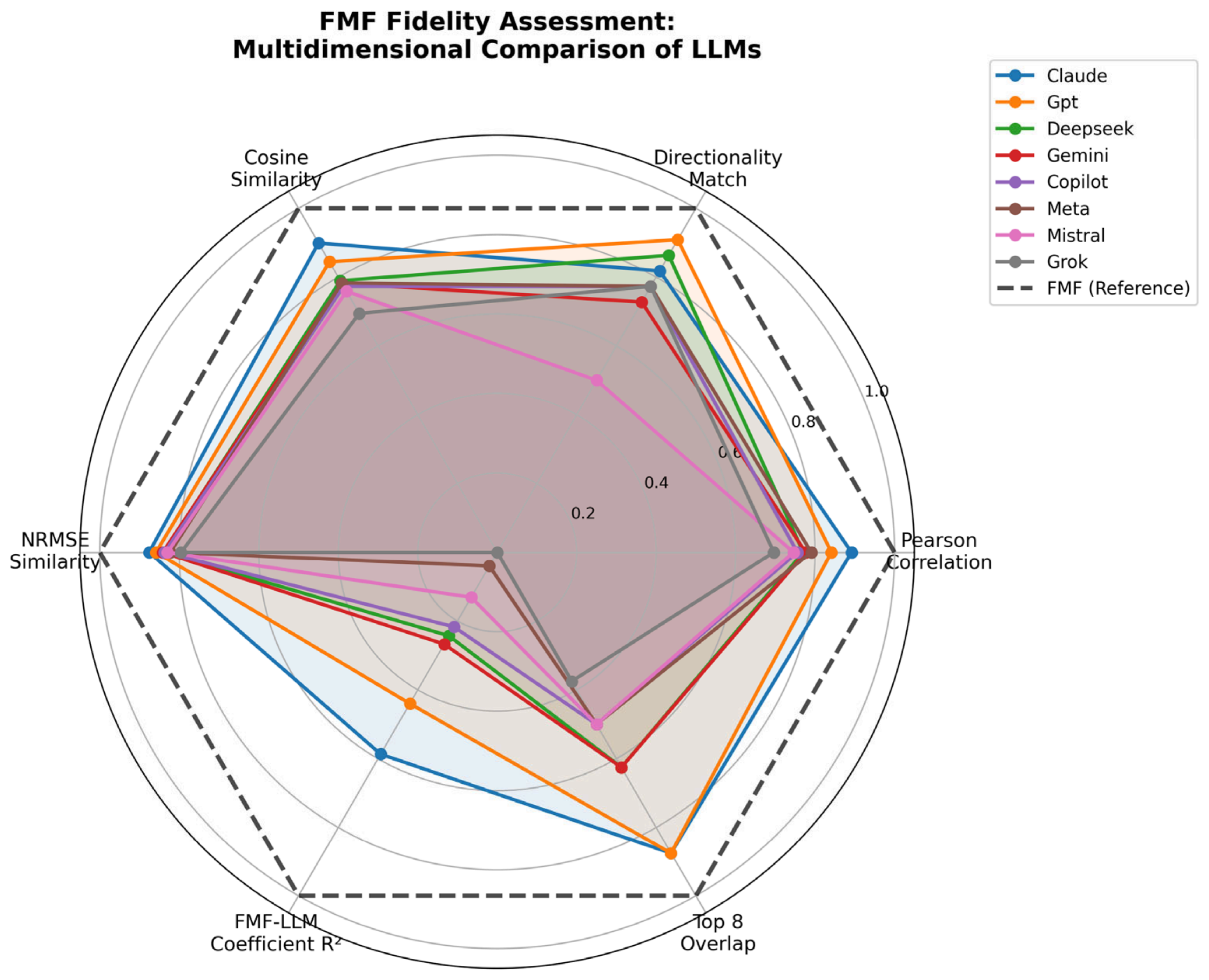
Multidimensional FMF fidelity assessment of LLMs across six evaluation metrics. Dashed line represents the FMF reference (perfect score on all dimensions). Larger enclosed areas indicate higher overall fidelity to the FMF model structure.

Figure 3 provides a multidimensional comparison across agreement metrics. Higher-performing models (Claude and GPT-5) showed more consistent agreement across multiple dimensions, whereas intermediate models preserved directionality with weaker magnitude alignment. Lower-performing models demonstrated reduced agreement across several metrics, particularly those reflecting magnitude and variance explained.

### 3.1. Comment

Directly querying large language models about preeclampsia risk factors often yields unstructured, prompt-sensitive responses that are not readily comparable quantitatively. To overcome this, we used a risk-based evaluation framework, conceptually analogous to failure mode and effects analysis, in which model behavior was inferred by systematically varying clinically relevant inputs and analyzing resulting changes in predicted risk.

Using this structured approach, this study provides the first systematic, quantitative comparison of how contemporary large language models weight established preeclampsia predictors relative to the FMF competing-risks model [8,9,20]. Applying local analysis [18] to 129 synthetic clinical scenarios, we observed variation in model alignment with FMF, with composite fidelity scores from 0.59 to 0.81 among eight models. These results suggest that some LLM outputs better approximate the local FMF predictor-effect structure than others; however, they do not establish the clinical reliability, predictive accuracy, or superiority of any model.

Importantly, this study does not evaluate clinical prediction performance, calibration, discrimination, patient outcomes, or clinical safety. Instead, it examines the extent to which LLM-generated risk estimates reproduce the local predictor-effect structure of a validated preeclampsia prediction model.

Three descriptive observations can be derived from this analysis. **First**, Claude and GPT-5 had the highest exploratory composite scores of 0.81 and 0.78 and shared 87.5% of the dominant predictors with the FMF reference. Because no formal statistical testing was performed, this should be interpreted as descriptive ordering rather than proof that one model outperforms another. **Second**, several models showed smaller local effect sizes than the FMF reference. This attenuation may reflect conservative probability scaling, prompt interpretation, or the fact that LLMs are not structured risk calculators, rather than necessarily indicating failure to recognize clinical associations. **Third**, lower-scoring models, particularly Mistral and Grok, showed limited coefficient-level agreement with the FMF reference in this local setting; this should be described as limited fidelity to FMF rather than fundamental misalignment with clinical evidence or proof of unsafe clinical behavior.

### 3.2. Model-Specific Performance Patterns

Claude showed the highest descriptive agreement with the FMF model, with correlation r = 0.78, cosine similarity 0.80, and explained variance R^2^ = 0.59. Its PLGF MoM coefficient (−0.41) and risk-factor weighting closely matched FMF values, reflecting strong placental biomarker representation. GPT-5 ranked second, achieving the highest directional accuracy (90.9%), correctly matching the risk direction for 20 of 22 predictors; however, differences in coefficient magnitude remained evident, as it tended to overestimate key placental and vascular markers like uterine artery PI and PlGF.

Other models showed lower or more heterogeneous agreement with the FMF reference. DeepSeek and Gemini generally preserved directionality but reduced local effect sizes; DeepSeek mostly ignored biochemical markers (PlGF and PAPP-A); and Gemini gave almost no weight to mean arterial pressure, one of the strongest FMF predictors [9,21].

Copilot and Meta demonstrated moderate agreement with selected coefficient inversions or weak variance explained: Copilot reversed effects for PlGF and PAPP-A, while Meta kept directionality but failed to match FMF’s effect structure (R^2^ = 0.04). Mistral and Grok had the lowest descriptive fidelity profiles, particularly for magnitude and explained variance. Mistral showed random accuracy, while Grok overlooked most FMF predictors and key first-trimester biomarkers.

### 3.3. Clinical Implications

Agreement with the FMF model should not be interpreted as evidence of clinical correctness or biological validity. The heterogeneous agreement observed across LLMs may be relevant for future patient-facing applications and clinical decision support, particularly as LLMs are increasingly used for pregnancy-related health information [10,22]. However, clinical implications cannot be inferred directly from this study, and whether these differences affect risk communication, decision-making, or outcomes requires dedicated clinical validation.

Differences in predictor representation were observed across models. Some LLMs assigned lower relative importance to prior gestational age at delivery than the FMF model, suggesting reduced emphasis on obstetric history in the local approximations. Isolated coefficient inversions for established biomarkers, such as PlGF or PAPP-A, also indicated disagreement in the direction of association for clinically relevant predictors [20,23,24].

More broadly, coefficient attenuation suggested that, even when directionality and rank order were partly preserved, the magnitude of predictor effects often differed from the FMF reference. This should not be interpreted automatically as clinical underestimation of risk, since it may also reflect conservative probability scaling, prompt interpretation, or the absence of a formal risk-calculator architecture in LLMs.

Rank-order agreement was generally higher than absolute agreement measures, suggesting that LLMs may better reflect the relative importance of predictors than their quantitative contribution to risk estimation. Taken together, these findings indicate variable alignment with established preeclampsia risk structures and support the view that LLMs should not replace validated clinical prediction tools. Their potential role may be limited to supportive functions, such as information summarization or communication, within a clinician-supervised framework.

Finally, this study evaluated only numerical risk estimates and derived feature-effect coefficients. It did not assess textual explanations, clinical recommendations, guideline adherence, hallucinations, medical consultation advice, aspirin prophylaxis recommendations, biomarker interpretation, or the appropriateness of risk communication. These aspects should be addressed in future clinical safety studies.

### 3.4. Comparison with Previous Literature

This study differs from prior evaluations that mainly assessed the factual accuracy, completeness, or qualitative adequacy of LLM-generated medical text [10,17,25,26]. Its main contribution is the use of an exploratory local perturbation and surrogate-model framework to compare numerical LLM risk estimates with a validated obstetrical reference model at the level of predictor effects. Therefore, the analysis evaluates structural similarity to the FMF competing-risks model, not clinical prediction accuracy or outcome-based validity.

Our findings are consistent with prior gynecologic evaluations showing that LLMs may provide broadly correct but incomplete information, particularly when nuanced clinical interpretation is required [17,25].

Recent AI studies in preeclampsia prediction have focused on supervised ML or deep-learning models trained on clinical, biomarker, electronic health record, or physiological data, with performance assessed using discrimination, calibration, validation cohorts, and clinical utility metrics [27,28]. In a Romanian first-trimester screening cohort, direct comparison of logistic regression, random forest, and XGBoost with FMF a priori and a posteriori risks showed that FMF outputs matched or outperformed the evaluated machine learning classifiers, particularly when biomarkers were included. This supports the role of the FMF competing-risks model as a strong clinical benchmark and reinforces the distinction between validated prediction systems and exploratory AI-output audits [29].

The broader explainable AI literature also emphasizes that post-hoc explanations require careful interpretation. SHAP (SHapley Additive exPlanations) provides a theoretically grounded additive feature-attribution framework, while LIME-based explanations may be sensitive to perturbation strategy, sampling, and model settings [30,31]. Accordingly, this framework should be viewed as a preliminary local audit of LLM behavior, not as a substitute for robust predictive validation, alternative explainability analyses, or clinical safety assessment.

## 4. Strengths and Limitations

This study offers several strengths. The local surrogate approach [18] allows direct comparison of feature-effect estimates with the FMF reference structure, addressing the difficulty of interpreting unstructured LLM responses [26,31]. Using controlled synthetic cases enables systematic evaluation of marginal predictor effects under standardized conditions [4]. Testing eight LLMs under the same prompt template provides a uniform experimental setting, and the multidimensional fidelity assessment provides detailed insights. However, these strengths apply to local structural comparison only and do not establish clinical ground truth or predictive validity. Focusing on first-trimester prediction further aligns with the optimal timing for aspirin prophylaxis [32].

Several limitations should be considered. First, local surrogate analysis provides local explanations around a defined reference case [18], and model behavior may vary across different baseline risk profiles, including intermediate- and high-risk pregnancies. The findings should therefore be interpreted as local approximations rather than global model behavior.

Second, the one-factor-at-a-time design deliberately removes predictor interactions, and the analysis therefore does not evaluate clinically realistic combinations of interacting risk factors.

Third, no formal robustness analysis was performed across alternative reference cases, neighborhood definitions, repeated perturbations, or different K-LASSO settings. We also did not compare the results with SHAP or other explanation methods. Consequently, the stability of the reported feature-effect coefficients remains uncertain.

Fourth, LLM outputs are sensitive to prompt formulation [14,33], and only a single prompt template was evaluated. Each scenario was queried once, using web interfaces with default provider settings, and temperature, sampling parameters, reasoning-mode settings, and backend version details were not uniformly available. The reported model ordering may therefore be influenced by prompt-specific behavior, stochastic response variability, or provider-side model updates.

Fifth, the analysis included only 22 predictors, limiting the robustness of correlation and regression statistics. No confidence intervals, bootstrap estimates, or formal pairwise significance tests were used to establish statistical differences between models.

Sixth, the study evaluated agreement in predictor structure rather than clinical performance. Absolute risk calibration, discrimination, sensitivity, specificity, decision-curve analysis, clinical utility, and patient outcomes were not assessed. A model could theoretically show coefficient-level alignment with FMF but still provide inaccurate absolute risk estimates; and conversely, coefficient disagreement does not directly prove clinical harm.

Seventh, the textual explanations, recommendations, and safety-related aspects of LLM responses were not analyzed. Therefore, the findings should not be used to infer suitability for patient counseling or clinical decision support.

Eighth, the FMF competing-risks model was used as a validated reference model for comparison because of its extensive validation and adoption. However, it is not an absolute ground truth. Discrepancies between LLMs and FMF reflect differences relative to this specific model and should not be interpreted as direct evidence of clinical incorrectness.

Finally, large language models are continuously evolving, and their behavior may change over time with updates in architecture, safety tuning, and deployment environment [10,33], though the methodology remains relevant. The findings therefore represent a time-specific evaluation and may not generalize to future model versions.

## 5. Conclusions

LLMs demonstrate heterogeneous and predominantly partial, prompt-specific alignment with the FMF competing-risks model in this local perturbation analysis. Models with higher descriptive fidelity scores preserved the direction and relative ranking of several key predictors, but agreement in the magnitude and scaling of predictor effects remained limited across all models.

These findings indicate that current LLM outputs can partially reproduce aspects of the predictor-effect structure embedded within a validated preeclampsia risk model, particularly with respect to directionality and rank ordering. However, the study does not evaluate clinical prediction accuracy, calibration, counseling quality, or patient safety. As such, LLMs cannot be considered substitutes for validated clinical prediction models in preeclampsia risk assessment.

The exploratory explainability-based framework presented in this study provides a structured approach for investigating whether artificial intelligence systems align with established clinical models at the level of predictor effects. This approach may apply to other domains in translational medicine where understanding model behavior relative to validated standards is essential.

LLMs are continuously evolving, and their performance and alignment with clinical standards may change over time. Ongoing evaluation using standardized frameworks is therefore necessary, along with further research to assess stability across clinical contexts, prompt variations, and model updates, and to explore whether alignment with validated risk structures can be improved.

## Figures and Tables

**Table 1:** Correlation, directionality, and magnitude agreement between large language models and the FMF preeclampsia model based on 22 predictors.

Model	Pearson Correlation ^a^	Spearman Rho	Directionality Match	Cosine Similarity	NRMSE Similarity	FMF Feature-Effect R^2 b^	Top 8 Overlap	FMF Fidelity Score ^c^
**Claude**	0.784	0.861	0.818	0.799	0.141	0.587	87.5%	0.81
**Gpt-5**	0.683	0.835	0.909	0.69	0.165	0.44	87.5%	0.78
**Deepseek**	0.541	0.678	0.864	0.579	0.191	0.243	62.5%	0.7
**Gemini**	0.552	0.589	0.727	0.563	0.188	0.267	62.5%	0.68
**Copilot**	0.511	0.517	0.773	0.546	0.194	0.217	50.0%	0.67
**Meta**	0.581	0.671	0.773	0.565	0.215	0.04	50.0%	0.64
**Mistral**	0.491	0.444	0.5	0.517	0.205	0.131	50.0%	0.59
**Grok**	0.393	0.491	0.773	0.389	0.257	−0.366 †	37.5%	0.59

Legend: ^a^ Pearson correlations were statistically significant for all models except Grok (*p* = 0.07). ^b^ FMF feature-effect R^2^ represents the proportion of variability in FMF feature-effect coefficients explained by the corresponding large language model coefficients. Higher values indicate closer agreement with the FMF model’s pattern and magnitude of predictor effects. ^c^ Metrics were linearly transformed to a common 0–1 scale solely for construction of the composite FMF Fidelity Score. † Raw regression-derived R^2^ for Grok was −0.366 and was truncated to 0.00 for descriptive comparability and inclusion in the composite score.

## Data Availability

The original contributions presented in this study are included in the article/Supplementary Material. Further inquiries can be directed to the corresponding author.

## Supplementary Materials

The following supporting information can be downloaded at: https://www.mdpi.com/article/10.3390/diagnostics16152340/s1, Supplementary Material S1. Synthetic Clinical Scenarios Used for Large Language Model Queries and FMF-Based Risk Assessment. Supplementary Material S2. Standardized prompt template used to query all evaluated LLMs together with synthetic clinical scenarios file. Supplementary Material S3. Mapping of the 16 clinical and biochemical risk factors included in the FMF competing-risks model to the 22 predictors used in the LIME-based feature-effect analysis. Categorical variables were expanded into binary indicator variables (one-hot encoding), while continuous variables were retained as single predictors. Supplementary Material S4. Python code used to calculate preterm preeclampsia risk according to the Fetal Medicine Foundation competing-risks algorithm. Supplementary Material S5. Python code used for local surrogate analysis and FMF fidelity assessment of large language model risk estimates.

## Abbreviations

FMF	Fetal Medicine Foundation
LIME	Local Interpretable Model-Agnostic Explanations
LLMs	Large language models
MAP	mean arterial pressure
MoM	multiples of the median
NRMSE	normalized root mean squared error
PAPPA	pregnancy-associated plasma protein-A
PlGF	placental growth factor
SHAP	SHapley Additive exPlanations
UtA-PI	uterine artery pulsatility index
