# Supplementary material for: Understanding of Preeclampsia Risk Factors in Large Language Models Compared with a Validated Competing-Risks Model
Source: Diagnostics (Basel). 2026 Jul 26;16(15):2340. doi: 10.3390/diagnostics16152340 (PMC13465115; doi:10.3390/diagnostics16152340)
Supplement: Supplementary file 1 [file diagnostics-16-02340-s001.zip › Supplementary Material S2.pdf]

Supplementary Material S2.

Standardized prompt template used to query all evaluated LLMs together with synthetic clinical scenarios file.

The following are the clinical, demographical, biophysical and biochemical characteristics of a {Age}-year-old pregnant woman. She is {Race}, has {Height} cm height and a weight of {Weight} kg. Her conception was {Conception}. She {Family_Hx_PE} family history of preeclampsia. She {Chronic_hypertension} chronic hypertension, {Diabetes_mellitus} diabetes mellitus and {SLE_APS} systemic lupus erythematosus or antiphospholipid syndrome. She is {Parity}, the interval between pregnancies is {Inter_pregnancy_interval_years} years and the last pregnancy ended at {Previous_last_birth_gestational_age} gestational weeks. At first trimester screening at 11 to 13 gestational weeks, her Mean Arterial pressure MoM was {Mean_arterial_pressure_MoM}, her Mean Uterine Arteries PI MoM was {Uterine_artery_PI_MOM}, her PLGF MoM was {Plgf_MoM} and her PAPPA MoM was {PAPPA_MoM}.

For each raw, complete the above template with risk factors according to their column name. Then, for all 130 patients, use the completed template to estimate the probability of preeclampsia before 37 gestational weeks, as a number between 0 and 1, with four decimal places. Show the case number and probability in a table with .csv format.
