# Supplementary material for: Understanding of Preeclampsia Risk Factors in Large Language Models Compared with a Validated Competing-Risks Model
Source: Diagnostics (Basel). 2026 Jul 26;16(15):2340. doi: 10.3390/diagnostics16152340 (PMC13465115; doi:10.3390/diagnostics16152340)
Supplement: Supplementary file 1 [file diagnostics-16-02340-s001.zip › Supplementary Material S3.pdf]

Supplementary Table S1. Mapping of the 16 clinical and biochemical risk factors included in the FMF competing-risks model to the 22 predictors used in the LIME-based feature-effect analysis. Categorical variables were expanded into binary indicator variables (one-hot encoding), while continuous variables were retained as single predictors.

FMF Risk Factor	Predictor Name (Used in Analysis)	Type	Description
Maternal age	Maternal age	Continuous	Age in years
Maternal race	Race: White	Binary	Reference category
	Race: Black	Binary	Indicator variable
	Race: East Asian	Binary	Indicator variable
	Race: South Asian	Binary	Indicator variable
	Race: Mixed/Other	Binary	Indicator variable
Maternal height	Maternal height	Continuous	Height in cm
Maternal weight	Maternal weight	Continuous	Weight in kg
Conception method	IVF conception	Binary	Assisted reproduction vs spontaneous
Family history of PE	Family history of PE	Binary	Yes vs no
Chronic hypertension	Chronic hypertension	Binary	Yes vs no
Diabetes mellitus	Diabetes mellitus	Binary	Pre-existing diabetes
Autoimmune disease	SLE/APLS	Binary	Systemic lupus erythematosus or antiphospholipid syndrome
Parity	Nulliparity	Binary	No previous births
	Multiparity without previous PE	Binary	Parous, no history of PE
	Multiparity with previous PE	Binary	Parous, prior PE
Interpregnancy interval	Interpregnancy interval	Continuous	Years
Previous pregnancy outcome	Previous gestational age at delivery	Continuous	Weeks
Mean arterial pressure	MAP MoM	Continuous	Multiples of median
Uterine artery PI	UtA-PI MoM	Continuous	Multiples of median
PAPP-A	PAPP-A MoM	Continuous	Multiples of median
PlGF	PlGF MoM	Continuous	Multiples of median
